# Beyond Hematuria: An Unexpected Diagnosis of Autosomal Dominant Polycystic Kidney Disease in a Young Adult Without a Family History

**DOI:** 10.7759/cureus.117085

**Published:** 2026-09-28

**Authors:** Souzan Moslehshirazi, Mauricio Chiriboga, Asya Starks, Salvatore Grasso, Peyman Arghavani

**Affiliations:** 1 Medicine, Ross University School of Medicine, Miramar, USA; 2 Internal Medicine, Dignity Health - California Hospital Medical Center, Los Angeles, USA

**Keywords:** autosomal dominant polycystic kidney disease (adpkd), hematuria, hypertension, premature closure, renal cysts, urinary tract infection

## Abstract

Autosomal dominant polycystic kidney disease (ADPKD) is one of the most common inherited renal disorders, yet its diagnosis may be delayed when initial symptoms overlap with more prevalent conditions such as urinary tract infection or nephrolithiasis. Premature diagnostic closure on these common etiologies can delay recognition of underlying renal disease, particularly in young adults without a known family history. We present the case of a 28-year-old man with no personal or family history of renal disease who was evaluated for gross hematuria. Renal imaging revealed extensive bilateral renal cysts. These findings, along with previously unrecognized hypertension and mild renal impairment, are consistent with ADPKD. This case emphasizes the importance of considering structural renal disease beyond common urinary etiologies in young adults presenting with unexplained hematuria and new-onset hypertension, even in the absence of a family history. Early recognition may prevent diagnostic delay, facilitate timely nephrology referral, and enable initiation of renoprotective therapy.

## Introduction

Autosomal dominant polycystic kidney disease (ADPKD) is the most common inherited kidney disorder, affecting an estimated 1 in 400 to 1 in 1,000 people worldwide [1,2]. Although typically autosomal dominant, family history is absent or unrecognized in a clinically important subset of patients: approximately 10%-25% of patients with ADPKD have no apparent family history, which may reflect de novo PKD1/PKD2 mutations, mosaicism, early parental death, or mild or unrecognized disease in an affected parent [3,4]. A negative family history should not, by itself, lower clinical suspicion.

ADPKD should be considered in patients with hypertension, flank or abdominal pain, hematuria, or recurrent UTIs and nephrolithiasis, particularly in a young adult with new renal impairment, as well as those with extrarenal findings such as hepatic cysts, cardiac valvulopathy, or intracranial aneurysms [1,5]. Because these features overlap heavily with far more common urinary conditions, clinicians are prone to premature closure [6]. Mutations in PKD1 (approximately 85%) or PKD2 (approximately 15%) disrupt polycystin-mediated ciliary signaling in renal tubular cells, driving progressive cyst formation and enlargement, with cyst-related vascular compression contributing to early activation of the renin-angiotensin system [5,7].

When family history is unclear, the differential broadens to include simple renal cysts, acquired cystic kidney disease, autosomal recessive PKD, tuberous sclerosis, and von Hippel-Lindau disease [8]. Renal ultrasound remains first-line, but the standard age-based diagnostic criteria are less reliable without a known family history, making genetic testing important for confirmation and subtyping in sporadic cases [2,9]. Management does not differ by family history: ACE inhibitors or ARBs remain first-line, with tolvaptan considered for rapid progressors [10,11]. Left unrecognized, ADPKD can progress to end-stage renal disease and cause hepatic, cardiac, and cerebrovascular complications, as well as cyst hemorrhage or infection [12-14].

## Case presentation

A 28-year-old man with no past medical history and no established primary care presented with one day of vague lower abdominal discomfort and three episodes of gross hematuria. He reported intermittent inguinal pain, raising concern for a urologic etiology such as nephrolithiasis, but denied flank pain or passage of stones. He denied dysuria, urinary frequency, urgency, suprapubic pain, or any aggravating or alleviating factors. He also denied recent fevers, chills, nausea, vomiting, or shortness of breath. The patient had no known family history of renal disease, intracranial hemorrhage, or autoimmune disorders, and denied any prior history of hypertension.

On initial evaluation, vital signs were notable for a blood pressure of 145/90 mmHg, which remained persistently elevated on repeat measurements throughout (148/92 mmHg, 145/95 mmHg, and 139/90 mmHg), consistent with sustained rather than transient hypertension. The temperature was 36.8°C, heart rate was 66 beats per minute, respiratory rate was 19 breaths per minute, and oxygen saturation was 98% on room air. Physical examination revealed a soft, non-tender abdomen without palpable masses or organomegaly, and no costovertebral angle tenderness. Initial laboratory studies demonstrated elevated serum creatinine and abnormal urinalysis findings, including hematuria and pyuria in the absence of bacteriuria (Table 1). Urine culture was obtained and returned no growth, making bacterial cystitis unlikely. STI was considered in the differential; however, nucleic acid amplification testing (NAAT) was not performed. The absence of urethral discharge, dysuria, and urinary frequency, combined with a urine culture with no growth and the subsequent imaging findings, made an STI an unlikely contributor to the clinical picture. Pyuria was presumed to reflect cyst-related inflammation rather than an infectious etiology.

**Table 1:** Initial laboratory and urinalysis findings at presentation

Parameter	Result	Reference range
Serum creatinine	1.5 mg/dL ↑	0.6-1.3 mg/dL
eGFR	66 mL/min/1.73 m² ↓	90 mL/min/1.73 m² or higher
Urinalysis		
Urine appearance	Hazy	Clear
Red blood cells, urine	>100 cells/high-power field (HPF) ↑	0-2 cells/high-power field (HPF)
White blood cells, urine	26-50 cells/high-power field (HPF) ↑	0-5 cells/high-power field (HPF)
Bacteriuria	Absent	Absent
Urine protein (Uprotein)	50 mg/dL ↑	0-14 mg/dL
Urine blood (Ublood)	Large	Negative
CBC		
White blood cells	5.6 109/L	4.5-11.0 × 10^9^/L
Red blood cells	4.92 million/mm^3^	Male: 4.3-5.9 million/mm^3^
Hemoglobin (Hgb)	14 g/dL	Male: 13.5-17.5 g/dL
Hematocrit (Hct)	40.40%	Male: 41%-53%
Platelets (Plt)	284	150-400 × 10^9^/L
General chemistry		
Sodium	140 mEq/L	136-146 mEq/L
Potassium	4.1 mEq/L	3.5-5.0 mEq/L
Chloride	111 mEq/L ↑	95-105 mEq/L
Glucose	99 mEq/L	Random, non-fasting: <140 mg/dL
Calcium	9.4 mEq/L	8.4-10.2 mg/dL
Magnesium	2.2 mEq/L ↑	1.5-2.0 mg/dL
BUN	19 mEq/L	7-18 mg/dL
BUN/Cr ratio	12.8	Between 10:1 and 20:1

Renal US (Figure 1) showed numerous cysts scattered throughout both kidneys, concerning for ADPKD. This finding was further investigated with CT of the abdomen (Figure 2), which revealed bilaterally enlarged kidneys with multiple cortical and medullary cysts of varying sizes, without evidence of hydronephrosis. The radiographic appearance, in conjunction with the patient’s age, elevated blood pressure, and elevated creatinine, made ADPKD the leading diagnosis. The patient was admitted to the internal medicine service for further evaluation and management. Nephrology was consulted.

**Figure 1:**
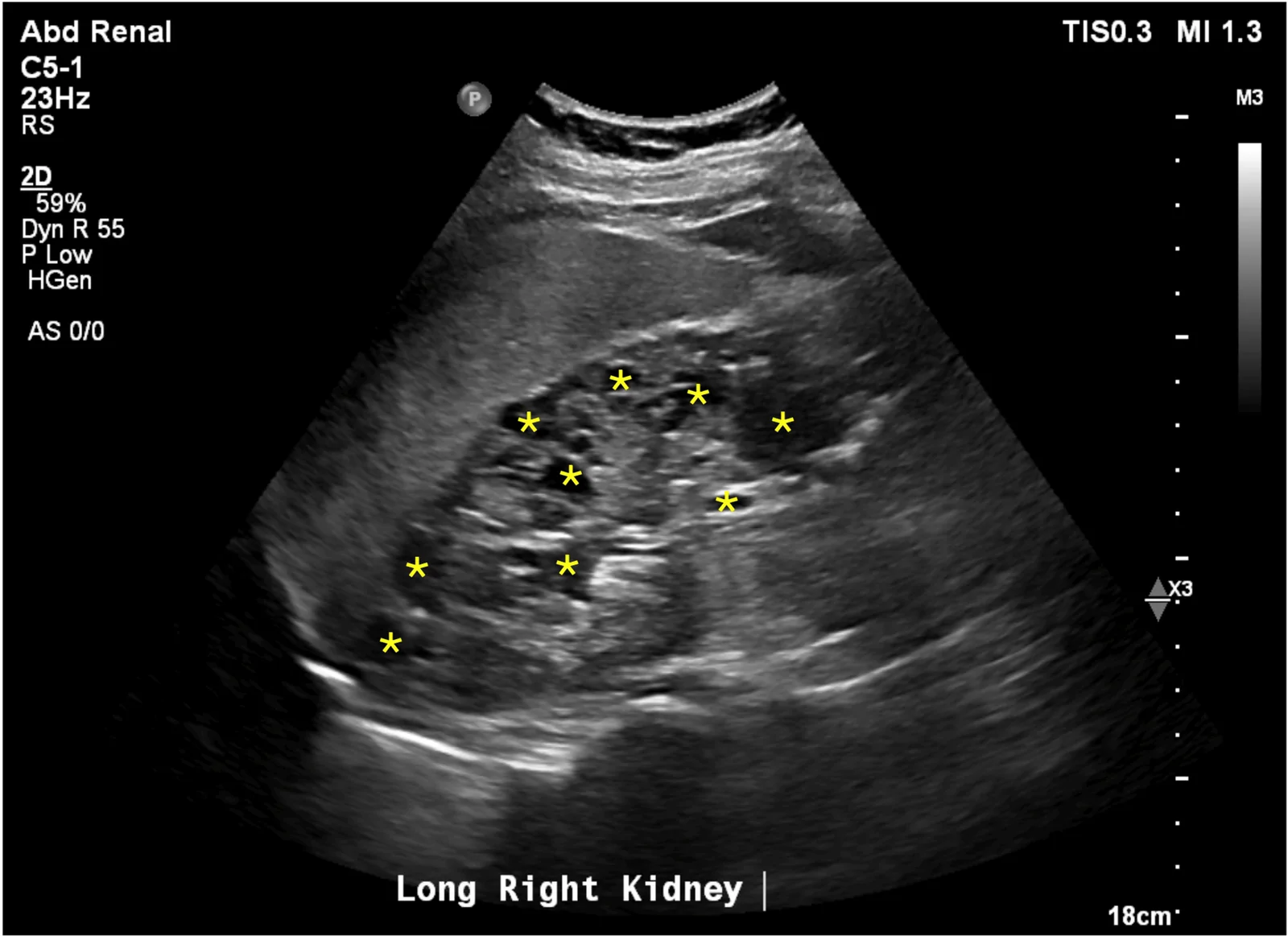
Abdominal ultrasound Yellow asterisks: cysts in the kidney.

**Figure 2:**
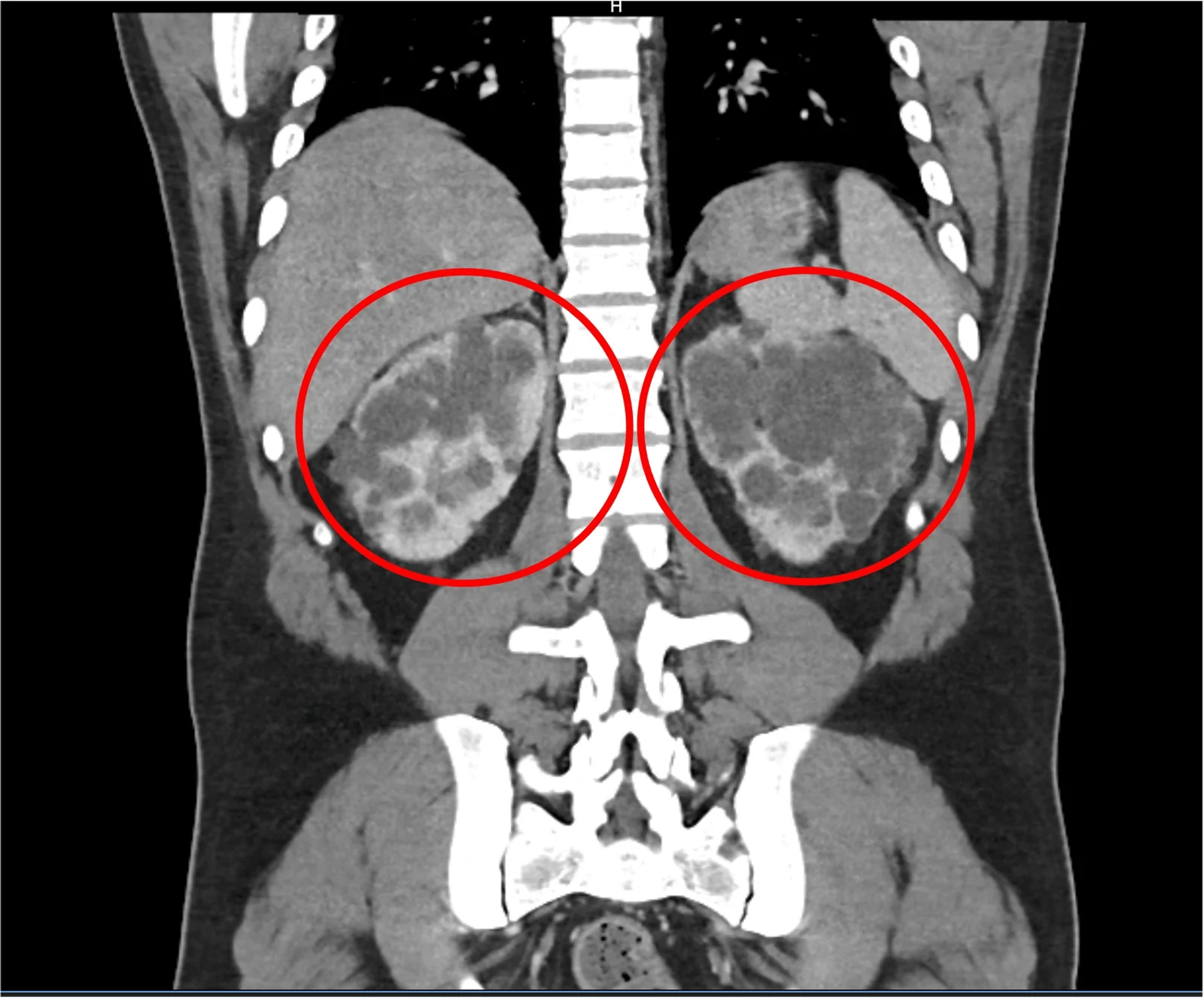
Non-contrast coronal CT of the abdomen Red circles: polycystic kidneys.

The patient was admitted for management of gross hematuria and hypertension in the setting of ADPKD. Blood pressure was initially managed with scheduled oral nifedipine and as-needed clonidine, and improved to 135/92 mmHg. Multidisciplinary discussion and risk assessment prompted evaluation of potential extrarenal manifestations. Given the known association between ADPKD and intracranial aneurysms, CT angiography of the head was performed and showed no aneurysms or acute cerebrovascular abnormalities.

The patient was subsequently transitioned to losartan 25 mg daily for long-term blood pressure control in alignment with guideline-directed management of ADPKD. Hematuria was attributed to cyst rupture and resolved without intervention. No evidence of cyst infection was identified during hospitalization, and antibiotics were therefore not warranted. Serum creatinine improved from 1.5 mg/dL to 1.3 mg/dL.

The patient was discharged in stable condition at the conclusion of hospitalization. He was counseled on home blood pressure monitoring, dietary sodium restriction, and adherence to prescribed antihypertensive therapy. He was also instructed on the importance of close nephrology follow-up.

## Discussion

This case illustrates two recurring challenges in the diagnosis of inherited renal disease in young adults: the risk of premature diagnostic closure toward infectious etiologies in the acute setting and the downstream consequences of absent preventive care.

Premature closure, defined as settling on a diagnosis before the full clinical picture has been considered, is among the most common cognitive errors in emergency and general medical practice and is a well-documented contributor to diagnostic delay across a wide range of conditions [6,15,16]. In acute care settings, abnormal urinalysis findings may bias clinicians toward infectious etiologies [17]. Here, the combination of hematuria and pyuria could reasonably have prompted empiric treatment for urinary tract infection (UTI) or, given the patient's age, evaluation for sexually transmitted infection (STI). However, the absence of dysuria, urinary frequency, pelvic pain, urethral discharge, and bacterial growth on urine culture reduced suspicion for either and prompted renal ultrasonography, which led to the diagnosis of ADPKD. Sterile pyuria, a recognized phenomenon in ADPKD attributable to cyst inflammation or microscopic cyst rupture, was considered the most likely explanation in this case [1,2].

The diagnosis was originally obscured by the absence of features classically associated with ADPKD, such as flank pain, palpable renal enlargement, and, most notably, family history. The absence of family history, as elicited by the patient, deserves particular emphasis. While autosomal dominant mutations in the PKD1 or PKD2 genes are most common, approximately 10%-25% of patients with ADPKD have no apparent family history, which may reflect de novo mutations, mosaicism, or previously unrecognized disease in an affected parent [2-4]. The patient denied relevant family history; thus, a sporadic mutation remains possible, although genetic analysis was not completed during the hospital stay. Reports of ADPKD presenting through symptoms that mimic common urinary conditions, rather than through incidental imaging or genetic screening, remain limited in the literature, underscoring how readily this diagnosis can be missed [3]. Maintaining a broad differential is, therefore, essential, especially in younger patients whose presentations may not follow classic patterns. The coexistence of gross hematuria, new-onset hypertension, and even mildly impaired renal function in a young adult should prompt evaluation for underlying renal disease, including renal imaging, rather than empiric treatment alone [18].

The presence of undiagnosed hypertension in this 28-year-old is the second key implication of this case, and it points to a gap in care that precedes the acute encounter. Hypertension in ADPKD, driven by cyst expansion and activation of the renin-angiotensin-aldosterone system, frequently develops well before significant renal function loss [7]. A blood pressure reading of 145/90 mmHg in this patient was not incidental and had very likely been present for some time. Starting losartan was consistent with current guideline recommendations favoring angiotensin-converting enzyme (ACE) inhibitors or angiotensin receptor blockers for their renoprotective properties [10]. Newly identified hypertension in a young adult, however, warrants a structured workup for secondary causes, which are more prevalent in this age group. Workup includes renal imaging for structural abnormalities such as polycystic kidney disease or renal artery stenosis, urinalysis and metabolic panel for parenchymal disease, aldosterone-to-renin ratio to screen for primary hyperaldosteronism, plasma or urine metanephrines to exclude pheochromocytoma, thyroid function testing, and assessment for obstructive sleep apnea [10]. Had this patient established routine primary care early in adulthood, a routine wellness blood pressure reading could have initiated this workup years earlier, potentially identifying ADPKD before significant cyst burden and renal impairment developed. Instead, like many young adults transitioning out of pediatric care, he encountered the health system only when acutely symptomatic. Acute care clinicians, therefore, have a unique opportunity with this population to look beyond the chief complaint and into years of missed interventions.

Further limiting the workup in our patient is self-reported lack of primary care, which elucidates the gap in care that can complicate all encounters. For clinician encounters with children or adolescents, blood pressure above the 95th percentile for age requires further workup, according to “The Fourth Report on the Diagnosis, Evaluation, and Treatment of High Blood Pressure in Children and Adolescents” [19]. Evaluation beyond a typical history includes kidney and serum assessment [19]. Young adult patients, according to the “Seventh Report of the Joint National Committee on Prevention, Detection, Evaluation, and Treatment of High Blood Pressure,” require assessment for secondary causes, beginning with evaluation of the kidney, particularly when hypertension presents under the age of 30 [20].

This case highlights how premature closure and limited access to preventive care intersect to compound diagnostic vulnerability. Hematuria and pyuria obscured the diagnosis by resembling more common urinary conditions, while the absence of routine preventive care delayed recognition of asymptomatic hypertension and underlying renal disease. Early recognition of ADPKD would have enabled a timely nephrology referral, risk stratification, initiation of disease-modifying therapy, and screening for extrarenal manifestations, including intracranial aneurysms [11-14]. While hypertensive management of this patient was consistent with current guidelines, an earlier diagnosis may have also facilitated longitudinal surveillance for cardiovascular complications beyond hypertensive etiologies, including nonischemic cardiomyopathies that have been described in patients with ADPKD [14]. Reframed in this way, this acute care encounter was not only an atypical presentation of gross hematuria, but also a reminder against cognitive bias and a visible manifestation of years of missed preventive care.

## Conclusions

ADPKD can present in ways that closely resemble common urinary conditions, making premature closure a diagnostic risk. This case highlights the need to maintain a broad differential in young adults presenting with gross hematuria, especially when newly discovered hypertension or renal impairment is also present. The absence of routine preventive care could have meant that hypertension in a 28-year-old went unrecognized for up to several years, delaying a diagnosis that a simple wellness visit could have identified. Additionally, in patients with a new ADPKD diagnosis, timely renal imaging, nephrology referral, and initiation of renoprotective therapy meaningfully improve long-term outcomes. 
